# Practicalities in running early-phase trials using the time-to-event continual reassessment method (TiTE-CRM) for interventions with long toxicity periods using two radiotherapy oncology trials as examples

**DOI:** 10.1186/s12874-020-01012-z

**Published:** 2020-06-22

**Authors:** Erik van Werkhoven, Samantha Hinsley, Eleni Frangou, Jane Holmes, Rosemarie de Haan, Maria Hawkins, Sarah Brown, Sharon B Love

**Affiliations:** 1 NKI, Amsterdam, Netherlands; 2 Cancer Research UK Clinical Trials Unit, Institute of Cancer Sciences, University of Glasgow, Glasgow, UK; 3 Clinical Trials Research Unit, University of Leeds, Leeds, UK; 4 MRC Clinical Trials Unit at UCL, UCL, London, UK; 5 Centre for Statistics in Medicine, NDORMS, University of Oxford, Oxford, UK; 6 CRUK MRC Oxford Institute for Radiation Oncology, Gray Laboratories, University of Oxford, Oxford, UK

**Keywords:** Phase I, Clinical trial design, TiTE-CRM, Late toxicity, Dose-finding, Adaptive trial design

## Abstract

**Background:**

Awareness of model-based designs for dose-finding studies such as the Continual Reassessment Method (CRM) is now becoming more commonplace amongst clinicians, statisticians and trial management staff. In some settings toxicities can occur a long time after treatment has finished, resulting in extremely long, interrupted, CRM design trials. The Time-to-Event CRM (TiTE-CRM), a modification to the original CRM, accounts for the timing of late-onset toxicities and results in shorter trial duration. In this article, we discuss how to design and deliver a trial using this method, from the grant application stage through to dissemination, using two radiotherapy trials as examples.

**Methods:**

The TiTE-CRM encapsulates the dose-toxicity relationship with a statistical model. The model incorporates observed toxicities and uses a weight to account for the proportion of completed follow-up of participants without toxicity. This model uses all available data to determine the next participant’s dose and subsequently declare the maximum tolerated dose.

We focus on two trials designed by the authors to illustrate practical issues when designing, setting up, and running such studies.

**Results:**

In setting up a TiTE-CRM trial, model parameters need to be defined and the time element involved might cause complications, therefore looking at operating characteristics through simulations is essential. At the grant application stage, we suggest resources to fund statisticians’ time before funding is awarded and make recommendations for the level of detail to include in funding applications. While running the trial, close contact of all involved staff is required as a dose decision is made each time a participant is recruited. We suggest ways of capturing data in a timely manner and give example code in R for design and delivery of the trial. Finally, we touch upon dissemination issues while the trial is running and upon completion.

**Conclusion:**

Model-based designs can be complex. We hope this paper will help clinical trial teams to demystify the conduct of TiTE-CRM trials and be a starting point for using this methodology in practice.

## Background

Discussion of model-based approaches to designing phase I trials has historically been limited to the statistical literature, focusing on theoretical properties of such designs and somewhat limiting their wider reach in applied health research. More recently however, continual reassessment method (CRM) designs for early-phase trials are beginning to be discussed outside of the statistical literature [1]. Papers focusing on application are also appearing [2], enabling these designs to become more accessible and more widely implemented.

The CRM design was originally described by O’Quigley and colleagues in 1990 [3], offering a model-based approach to dose escalation decision-making based on an underlying dose-toxicity relationship. There are a number of published adaptations to this original CRM design including the time-to-event continual reassessment method (TiTE-CRM) [4]. The TiTE-CRM may be applied to settings where dose limiting toxicities are expected to occur beyond a typical observation period of a few weeks. This is particularly relevant to the field of radiotherapy, where toxicities can often occur up to and sometimes longer than 6 months after treatment [5]. With the TiTE-CRM design, participants who have not completed their toxicity period contribute to the calculation of the next participant’s dose allocation, weighted by the proportion of the toxicity follow-up that they have completed. Participants can be continually recruited, reducing the overall duration of the trial, and all information is used to assign new participants to the best dose [6].

The implementation of a TiTE-CRM trial includes complexities such as defining model parameters, putting allocation and stopping rules into place and ensuring resources are in place for timely data collection and reporting. Many of these issues relate to the time aspect and are therefore novel to this modification. Clarity of these issues comes with use but they are, initially, challenging with the current literature.

We discuss the practicalities of designing, setting up, and running TiTE-CRM trials, from grant application to dissemination, using two recent phase I radiotherapy oncology trials as examples.

## Methods

The CRM encapsulates the dose-toxicity relationship in a statistical model. This model uses all available data from previously enrolled participants to determine the next participant’s dose and subsequently the maximum tolerated dose (MTD). The CRM requires all participants currently on the trial to be followed up for the entire observation window before their data can be used to estimate the next participant’s dose [2]. This is not always feasible in practice, especially if the observation period is very long, such as in radiotherapy trials.

The TiTE-CRM is a modification of the original CRM developed to address the issue of late-onset toxicities [4]. In addition to those participants who complete follow-up or experience a toxicity, it accounts for participants who have not been followed up completely. Data are weighted according to how much information each participant provides. The resulting weighted dose-toxicity model incorporates both fully and partially observed participants. When designing a trial using the TiTE-CRM, we define the following parameters:

- A maximum sample size of *N* participants to be recruited;
- A target toxicity level, *TTL*, denoting an acceptable probability of dose-limiting toxicity (DLT);
- *K* dose levels to be explored, labelled *d*_1_, …, *d*_*K*_;
- A DLT observation time period of length *T*, also called the DLT window;
- An increasing sequence of prior estimates of the DLT probability at each dose, also called the skeleton, ***π***_**0**_ = {*π*_01_, …, *π*_0*K*_};
- A functional form for the dose-toxicity curve, for example, the power function $\mathrm{F}\left({d}_k,\beta \right)={d}_k^{\exp \left(\beta \right)}$, with k = 1,2 … .K; and
- A prior distribution for the model parameter(s) of the dose-toxicity curve, for example, a normal distribution for the parameter of the power function (*β*): *p*(*β*) = *N*(0, *σ*^2^)

At the start of the trial, dose labels *d*_1_, …, *d*_*K*_ are calculated by solving F(*d*_*k*_,  *E*(*β*)) = *π*_0*k*_ where *E*(*β*) is the prior mean of *β*. This choice of the dose labels guarantees that at the start of the trial the model fits the probabilities given by the prior estimates, ***π***_**0**_ . After each participant is recruited, these estimates are updated to the posterior estimates.

Suppose there are *J* participants currently enrolled on the trial. The available information is the set of doses {*x*_1_, …, *x*_*J*_} administered to the *J* participants and the set of toxicity outcomes.

{*y*_1_, …, *y*_*J*_}, where *y*_*j*_ = 0 and *y*_*j*_ = 1 denote the absence or presence of a toxicity event for participant *j*, respectively. Each participant has been observed for an amount of time {*t*_1_, …, *t*_*J*_}, where 0 < *t*_*j*_ ≤ *T*.

Each participant is assigned a weight, *w*_*j*_, which is a function of their follow-up time unless and until they have a DLT, when they are assigned full weighting. The most commonly used weight function is:

$$ {w}_j\left({t}_j;T\right)=\left\{\begin{array}{c}\frac{t_j}{T},\kern0.5em {y}_j=0\\ {}1,\kern0.5em {y}_j=1\end{array}\right., $$

which is linear in the follow-up time, *t*_*j*_, until the end of the observation window, *T*. This choice of weight function is reasonable if little is known about the DLT incidence in time, or if the incidence rate is approximately constant up to time *T*. Cheung et al. discuss other weight function options [4] and we give an example in the Additional file 1.

The TiTE-CRM model uses a weighted likelihood function to calculate the posterior mean of the model parameter(s) (one parameter, *β*, in this example) after the evaluation of *J* participants, given by

$$ {L}_J\left(\beta \right)=\prod \limits_{j=1}^J{\left[F\left({x}_j,\beta \right)\ast {w}_j\right]}^{y_j}{\left[1-F\left({x}_j,\beta \right)\ast {w}_j\right]}^{1-{y}_j} $$

For each dose *k*, the plug-in estimate of the toxicity probability is then calculated using ${\hat{\beta}}_J$, the posterior mean of *β*, as follows:

$$ {\hat{\pi}}_k=F\left({d}_k,{\hat{\beta}}_J\right)={d}_k^{\exp \left({\hat{\beta}}_J\right)}. $$

**Fig. 1:**
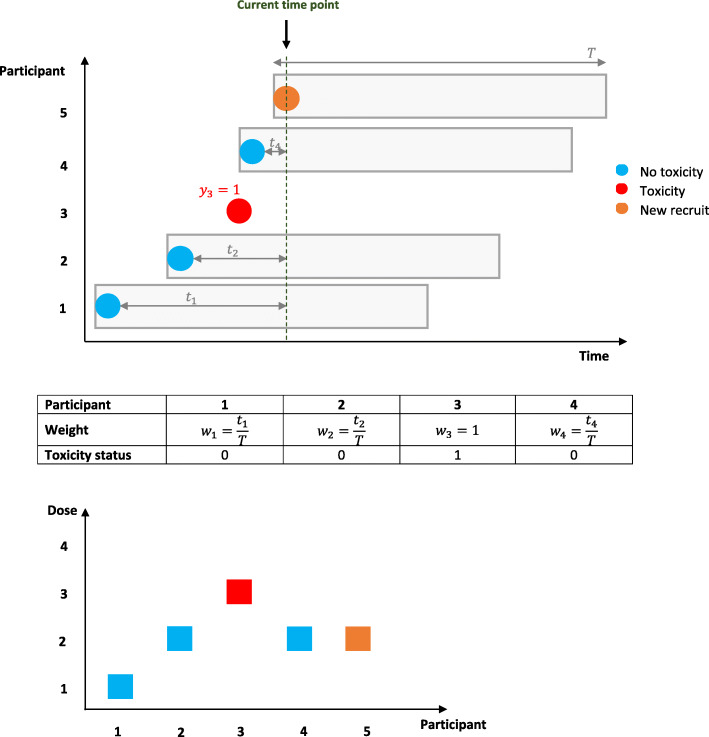
Graphic illustration of the TiTECRM method. The first plot shows the recruited participants over time. We illustrate the observation window ***T*** for each recruit; ***y***_**3**_ has experienced a toxicity hence omitting presenting the full observation window. The dose allocated for participant 5, at the current time point, is decided by accounting for all of the available data, which includes the toxicity status and weights of participants 1–4. These will be accounted for in the calculation of the updated dose-toxicity curve. The table shows the weight that each participant contributes in updating the dose-toxicity curve when participant 5 is recruited onto the trial. Although participant 3 has not completed the observation window, they have experienced a toxicity event, so their contributed weight to the model is 1. The second plot presents the dose allocation for each participant. In this scenario, once a toxicity is observed, the model recommends de-escalating to dose level 2. As participant 4 was not on the trial for long when participant 5 was recruited, the model recommends the same dose for participant 5. This figure reflects the example code and (fictitious) data presented in the Additional file 1

The MTD is defined to be the dose level *k*^∗^ such that ${\hat{\pi}}_{k^{\ast }}$ is maximised but remains below the target toxicity level, *TTL*, or is the closest to the *TTL*, depending on the definition used. When a dose decision is reached, the current best guess for the MTD is calculated based on all data accrued so far. The trial continues to recruit participants until one of the stopping rules is satisfied (for example, the maximum sample size is reached) and the MTD is declared. Figure 1 shows how the method works graphically.

A comparison of the TiTE-CRM and the rolling six design [7] (a rule-based design allowing 6 at a dose level) concluded that the TiTE-CRM was superior as it treated all available participants, identified the MTD more accurately, and did not increase the probability of exposing participants to toxic doses. Similarly the TiTE-CRM has been compared with the 3 + 3 design and was found to be superior in its performance [6].

Table 1 gives some examples of published trials using the TiTE-CRM.

**Table 1:** Examples of clinical trials designed using the TiTE-CRM

Publication	Trial details
Ben-Josef et al. [8]	Radiation delivered with gemcitabine in participants with unresectable pancreatic cancer
Brown et al. [9]	Concurrent temozolomide and intensity modulated radiation therapy in glioblastoma multiforme
Chugh et al. [10]	Doxorubicin plus cixutumumab in soft tissue sarcoma
Frangou et al. [11]	Radiotherapy, chemotherapy, and M6620 in participants with oesophageal cancer (CHARIOT)
Kim et al. [12]	Whole brain radiotherapy and RRx-001 in radioresistant melanoma brain metastases
Kyriakopoulos et al. [13]	APC-100 in male participants with castrate-resistant prostate cancer
Haan et al. [14]	Three trials of radical radiotherapy and Olaparib in non-small cell lung cancer, breast cancer, and head and neck squamous cell carcinoma
Lao et al. [15]	Bortezomib administered in combination with whole brain radiotherapy in participants with brain metastasis
Lepeak et al. [16]	Continuous MKC-1 in participants with advanced or metastatic solid malignancies
Muler et al. [17]	Cisplatin combined with gemcitabine and radiation therapy in pancreatic cancer
Schneider et al. [18]	Vorinostat in combination with docetaxel in participants with advanced and relapsed solid malignancies
Tevaarwerk et al. [19]	Continuous MKC-1 in participants with advanced or metastatic solid malignancies
Zhen et al. [20]	Cabozantinib and gemcitabine in participants with advanced pancreatic ductal adenocarcinoma

To illustrate the practical implementation of the TiTE-CRM we use two TiTE-CRM trials designed by the authors. CHARIOT (ClinicalTrials.gov Identifier: NCT03641547) is a phase I, dose-finding trial using the TiTE-CRM framework that is currently open to recruitment in the United Kingdom [11]. It is the first trial examining the combination of radiotherapy, chemotherapy, and the ataxia telangiectasia mutated Rad3-related (ATR) inhibitor M6620 in participants with oesophageal cancer. The trial aims to identify the maximum tolerated schedule associated with no more than a predefined TTL, using a DLT assessment window of 24 weeks.

Our second example is the Olaparib and Chemoradiotherapy in Locally Advanced NSCLC trial (ClinicalTrials.gov Identifier: NCT01562210) [14]. This trial started off as a 3 + 3 design to investigate the safety of the PARP inhibitor olaparib in selected participants with non-small cell lung cancer (NSCLC). During the course of the trial the design was changed into a TiTE-CRM design. Participants were treated with chemo-radiotherapy, either concurrent or sequentially, at the discretion of the multidisciplinary tumour board. These treatment arms were considered separate groups for the purpose of estimation of the MTD of olaparib. The trial was chosen as an illustration here because it used an atypical function for the observation weights. The DLT time window of 1 year was divided into an acute-DLT period of 3 months, which was assigned half of the total weight. The remaining 9 months were assigned the other half of the weight, so that the weight function was piecewise linear over time. The rationale for this choice of the weight function was that the DLT definition comprised toxicities that are known to occur early (for example haematological toxicities) and toxicities that occur later (for example pneumonitis) and that their frequency of occurrence would be roughly equal. Details of the trial design will not be shown here, but the weight function is used as an illustration in the Additional file 1.

We use our experience with the TiTE-CRM design to discuss practical issues when designing, setting up, and running trials using this methodology. Extensions and modifications of the TiTE-CRM methodology have been proposed but are not discussed here [21–24].

## Results: trial design/pre-trial decisions

There are many decisions to make when designing a TiTE-CRM trial. As with a CRM design, the number of doses, the target toxicity level, the shape and skeleton of the dose-toxicity curve, method of inference, escalation rules, maximum sample size, and cohort size must be decided [2]. Decision rules should be put in place for stopping the trial early if the treatment is too toxic and for situations when the trial may be stopped early if the MTD is certain enough. A TiTE-CRM design poses extra questions for some of these common issues and adds other design decisions.

The length of the DLT window is a key question for the clinical experts on the trial team. This is the follow-up period from the start of treatment and needs to be long enough to encompass late-onset toxicities. Experience with the treatments and any literature in the same disease and treatment setting should help define the DLT window.

The prior probability of the DLTs, at each dose level, needs to be determined. One useful starting point is the estimated probability of DLT without the experimental treatment (i.e. a certain ‘background DLT probability’, e.g. the incidence of grade 3 pneumonitis after concurrent chemoradiotherapy for NSCLC without olaparib). Ideally, this is based on clinical data using the same radiotherapy scheme and technique. All dose levels should have a prior DLT probability at or above this estimated ‘background DLT probability’. To estimate the additional DLT probability due of the addition of an experimental drug, a model of the relationship between the radiation dose and the probability of toxicity can be used. Such models are known as normal tissue complication probability (NTCP) models in the field of radiation oncology [25]. The effect of the addition of the experimental drug can then be approximated by multiplication of the radiation dose in a NTCP model with dose enhancement factors obtained from preclinical studies. As DLT usually consists of several types of toxicity, for example depending on the organs at risk in a radiotherapy trial, determining a ‘background DLT probability’ and estimating the additional DLT probability should ideally be performed on all of these toxicity types.

Although the considerations for deciding the dose-escalation rules and cohort size are broadly the same as for the CRM, the TiTE-CRM’s time element introduces complications. We need to consider how many participants are recruited to a dose level and the amount of follow-up required before escalating to a higher dose. For instance, a trial may choose to follow one participant for the full DLT window before allowing escalation. This decision can affect the cohort size, as larger cohorts can result in more information on the tested doses, and is linked with deciding whether to pause recruitment while waiting for enough information to escalate the dose.

Looking at best- and worst-case scenarios can help to decide whether to pause recruitment. Assume that all participants who have not yet completed follow-up have a DLT in one scenario, and none have a DLT in the other scenario. If the dose decision is the same in both scenarios, then there is nothing to be gained by pausing recruitment. A simple accrual suspension rule, using a decreasing linear function of follow-up time, has been proposed by Polley et al. [26] Bekele *et al* [27] have proposed an alternative dose-finding method that uses predicted probabilities to suspend accrual if the risk of toxicity for future participants is too high. It is essential to perform simulations to assess how the planned trial would work in practice and ensure the study will be both safe and accurate. The results of these simulations, known as operating characteristics, include the probability of selecting the correct dose and the average number of patients and toxicities observed at each dose level. Simulations should consider accrual rates and recruitment pauses, cohort size, and information required for escalation, to determine the optimal design. The operating characteristics in CHARIOT included the proportion of times each schedule was the recommended schedule at the end of the trial, the proportion of patients treated at each schedule, the percentage of DLTs observed and the average trial size [11]. Software for these simulations is available in R (package dfcrm [28]) and SAS (see Salter [29] Table 1), although extra code may be needed for a specific trial.

The definition of an evaluable participant and the participant population to be included in dose decisions needs to be decided. For safety, an evaluable participant is generally any participant that has received any treatment. For dose-escalation, there may be a minimum amount of treatment that must be received to enable escalation in future participants, below which the participant may be replaced. If the participant is not replaced, a plan for how they will be included in the analysis is needed, perhaps via weighting. The participant may be included in the analysis even if they are replaced. This issue is most pertinent to dose-finding trials where treatment is given over a period of time. For instance, consider a participant who has been followed for the full DLT window without a DLT, but who only received two-thirds of the planned treatment. Before starting the trial, a decision needs to be made on how to include this data. CHARIOT uses cohorts of one participant, except at the start where three participants are treated and fully followed up before escalation is allowed. CHARIOT does not currently define an evaluable participant. Instead, the trial management group can take the treatment received into account when making a dose escalation decision and call on the independent data safety and monitoring committee for input. In the olaparib NSCLC trial, no formal definition of evaluability was given, but participant weighting was adjusted according to whether at least 80% of the olaparib dose was received. For participants who received less than 80% of the dose and did not develop a DLT, their weighting was calculated accounting for follow-up until the last day that full olaparib treatment was received, resulting in a reduced weight. In combination with the restriction that at least three participants must have been exposed for a minimum 3 months each before a dose escalation was allowed, this meant that an extra participant would be treated on that dose. Thus there was no need for a formal replacement rule. If a DLT occurred at any point (even after the 3-month period), such participants would be counted with full weight, to prevent the dose-toxicity curve from becoming overly optimistic.

## Results: Grant issues

One key issue with all model-based phase I designs is the amount of time required at the trial design stage, before grant funding is received [19, 30]. This problem is exacerbated when using TiTE-CRM due to the extra complexities of the trial design and set-up. The design and set-up of a team’s first TiTE-CRM trial will take several months and require both clinical and statistical expertise. The learning curve is less pronounced if a statistician on the team has experience designing a CRM trial, but the additional requirements of the TiTE-CRM will still realistically take weeks. Each aspect must be simulated and the impact of each component discussed with the clinical team.

The biggest challenge with the amount of time required to design a TiTE-CRM study, particularly for academic trials units, is how to fund the statisticians’ time during this period. Although this is a consideration for all clinical trial grant applications, TiTE-CRM designs require vastly more time before grant funding. Some options for dealing with this gap in funding include:

- Using infrastructure funding, if available (not all academic trials units have access to such funding);
- Applying for funding through a separate grant (for example from a disease-specific charity);
- Covering the cost through other grant funding and recouping these costs once the trial is funded (unfunded trials will result in a deficit);
- Incorporating time into the grant application for designing and setting up the TiTE-CRM model and writing into the application what will be taken into account in the design set-up. This approach should be discussed with the proposed funder early to ensure that it is acceptable, as it may affect how quickly the first participant is recruited after funding is approved.

The detail required in a grant application for a TiTE-CRM trial is dependent on the specific funder and is likely to change as model-based phase I trial designs, including the CRM and TiTE-CRM, become more widely understood. Table 2 lists our recommendations of the minimum information that should be included, based on our experiences. As the TiTE-CRM design is so complex, it may be necessary to provide minimal information in the main grant application and include the remainder in an appendix. We highly recommend early discussions with the proposed funder to determine what they expect to see in the grant application and to gain an understanding of their knowledge of model-based phase I trial designs.

**Table 2:** Minimum recommended detail to be included in grant applications. X shows the application stage where the information is required

Information to be included	Outline (for two-stage applications) or full (where the grant application requests funding and time to design and set-up the model)	Full (where the grant application does not include time to design and set-up the model)
Reference to key TiTE-CRM literature and a brief explanation of why this design is being used, as reviewers may not have encountered it before.	X	X
Sample size. If this is not fixed, provide an upper and lower bound.	X	X
	If not confirmed, add a note to say it will be confirmed after further simulations have been undertaken.	
Dose-limiting toxicities	X	X
Target toxicity level	X	X
		Include justification and how this was determined.
Dose-toxicity curve		X
Number of dose levels	X	X
	Include an estimate if this is not yet known.	
Starting dose level		X
Stopping rules		X
Any restrictions on recruitment or dose escalation		X
Software or packages used to set up the model and perform simulations		X
Information on simulations to be performed	X	
	Include details of toxicity timing and recruitment rates	
Simulation results		X
		Include details of toxicity timing and recruitment rates
How the data will be used throughout the trial to determine dose decisions		X
		Discuss the role of the safety review committee and how late toxicities will be incorporated in the trial. Explain that dose decisions are not made solely by the TiTE-CRM model.

## Results: running the trial

One of the challenges of a CRM design, whether with a time-to-event endpoint or not, is that close collaboration is required between clinicians, data managers, and statisticians during the trial. The dose-toxicity model must be re-estimated every time a dose estimation is performed. The dose and toxicity data therefore need to be updated regularly. This issue is even more important in a TiTE-CRM trial because participants can be included in the trial at any time.

Data can be collected on case report forms (CRFs), but in our experience, data are sometimes entered after considerable delays. To mitigate this, the Netherlands Cancer Institute uses a small database to capture only the minimal information required for calculating the next dose level. The CHARIOT study puts the essential data for dose escalation decisions into one CRF and ensures that sites complete this CRF before dose escalation meetings/decisions.

We recommend putting a standard operating procedure (SOP) in place that describes the roles and responsibilities of staff involved. Some suggested roles are:

- **Clinician:** ensure and verify that up-to-date data are provided via the route decided by the trial team, such as a small database or simple CRF;
- **Statistician:** perform the dose calculations and archive the data extracts, computer code, and results of each calculation so that they are available for inspection by reviewers and regulators;
- **Trial monitor/coordinator:** perform source data verification (SDV).

The roles of other personnel should be added as required.

Clinical trial monitors and coordinators at our institutions perform SDV at different times. Some do so before a dose escalation decision and others after a dose escalation decision, but before the next one. A process should be in place to ensure that the data used to perform dose calculations are accurate. However, some of our institutions make data accuracy the responsibility of the trial investigators, not the trial monitor or coordinator. In an ideal situation with sufficient resources, the clinical trial monitor would perform an SDV independently of the investigators before a decision without slowing down the trial. The ICH E6 (R2) good clinical practice guideline [31] currently makes no recommendations about this issue.

To prevent aggressive escalation early in a trial, it is recommended that the first few participants in the trial (for example, the first three) are treated with the starting dose [4]. A computer program must be prepared to estimate the dose-toxicity curve’s parameter (β) before the next participant enters the trial. Several software packages are available. Our preferred software is the R package dfcrm [28] as it uses open-source software, and additional packages for R make it easy to reproduce the calculations.

We give an example of a script in an R-markdown file that can be used as a template and to create a .pdf output in Additional file 1. The first part of the script loads a data file that contains (fictitious) information for calculating the next dose level. In the example, we import the data as a text file, which can be produced by most database programs, including Excel. The follow-up is calculated in days since the treatment began and printed to the output for verification. Parameters are specified and a custom weight function is defined. The weights are calculated and the function *titecrm* is called. Its output is presented in the resulting pdf file.

Additional restrictions on escalation to a higher dose are recommended. Commonly used restrictions include not allowing any doses to be skipped in the escalation scheme and requiring a minimum amount of cumulative exposure time on a previous dose level before escalation. In the example in the Additional file 1, doses cannot be skipped and at least three participants (not necessarily consecutive) need to have been exposed for a minimum 3 months each before escalation to a higher dose is allowed. It is common to use participant recruitment slots to prevent too many participants from being recruited early in the trial or just after a dose escalation, because more information needs to be accumulated on the current participants in the trial before a new participant is admitted [11]. These restrictions must be described in the protocol during trial design.

Even with restrictions in place, the dose level recommended by the model should never be followed blindly. It is difficult to simulate all of the possible trial scenarios during design. For example, new evidence may be discovered outside the trial. The investigators can choose not to escalate to a dose when the model suggests it, which occurs more often in TiTE-CRM than CRM due to the time element. It may even be appropriate to reduce a participant’s dose during the trial. For example, if many DLTs have occurred at a particular dose level and it becomes clear that the MTD will be lower, then the dose of other participants still on that dose level could be lowered. In this scenario, thought would need to be given as to how to include these participants in the analysis. Another relevant issue is the consideration of an emerging toxicity profile not included in the dose limiting toxicity definition. For example, grade 3 dysphagia during concurrent chemoradiotherapy for NSCLC is seen frequently and therefore usually not considered a DLT. However, if all participants at a particular dose level develop grade 3 dysphagia this dose level might be considered to be above the MTD. Ideally, the study team pre-defines such additional maximal accepted toxicity rates, however, as stated before, it is difficult to simulate all of the possible trial scenarios during design. Therefore, while running the trial, the study team should assess the emerging toxicity profile beyond the DLT incidence when approving recommended dose escalation by TITE-CRM. Clinical review should always guide the final dose escalation decision.

## Results: dissemination

During the trial, clinicians will know the current best estimate of the MTD. Care must be taken when releasing this information more widely. Although this is true for most phase I designs, it is particularly important for TiTE-CRM trials as participant follow-up may not be complete. If the MTD is released, the credible interval must also be given.

The data and results from a completed TiTE-CRM trial can be reported in the same way as for a CRM trial or any other model-based phase I trial. It should be made clear that the TiTE-CRM methodology has been used, and the methodology and any enforced recruitment restrictions should be adequately described.

## Discussion

Although model-based phase I trial designs, particularly the CRM, are being discussed more widely, many of these designs do not work well in practice when late-onset toxicities are present, such as in the field of radiotherapy. There are a growing number of papers discussing ways to improve the evaluation of radiotherapy treatments in phase I clinical trials, some of which make reference to the TiTE-CRM [5, 32, 33]. The TiTE-CRM allows for continual recruitment and includes all recruited participants when deciding the best dose for the next participant(s), weighted for the amount of follow-up that has been observed.

We have outlined the methods behind the TiTE-CRM. Based on our experiences, we have offered examples and advice on how to set up a study using this methodology, apply for funding, run it, and disseminate the results.

Setting up a TiTE-CRM trial requires decisions about dose escalation rules, cohort sizes, and follow-up that go beyond the considerations of a CRM trial without the time-to-event component. Performing adequate simulations to determine the impact of these considerations before finalising the design is vital.

Designing a TiTE-CRM trial is time-consuming, particularly the first time a clinical trials unit uses this methodology. We have outlined some options for funding statisticians’ time to cover this lengthy design period. As the TiTE-CRM and similar designs become more widely used, the information required at each stage of a grant application may change, and is likely to vary between funders. We therefore cannot provide a definitive list of what should be included in a grant application, but instead have recommended the minimum information to include at each application stage. We recommend discussing the trial with the proposed funder before applying to understand what they expect in the grant application.

Close collaboration between all members of the trial team is required once any phase I trial opens. This is particularly true for TiTE-CRM trials as participants can be included in the trial at any time, and the model is updated based on data at the current time-point, rather than on data that have already been collected. We have suggested ways to ensure that the data are up to date and recommend that a standard operating procedure is put in place to outline the roles and responsibilities of everyone involved in the trial. We have provided (Additional file 1) and explained (“Results: Running the trial”) example code for updating the dose-toxicity curve and determining the recommended dose for the next participant(s). Although a TiTE-CRM trial is set up based on many simulations of different scenarios, it is difficult to envisage all of the possible situations that can be encountered during the trial. The model recommendation should therefore never be followed blindly. The final decision of what dose each participant gets should be guided by both clinical judgement and the model recommendation.

The TiTE-CRM methodology has limitations, many of which are discussed in Sharon et al. [30] A lot of statistical time is needed when designing the study, which can be hard to find funding for. Much input is required throughout the study from the clinical, data management, and statistical teams, and many meetings with a safety review committee are required. Although TiTE-CRM speeds up a phase I trial with a long DLT observation window, it may not be ethical or safe to have too many participants on the trial at any one time, and it is not sensible to enter too many participants at a low dose. The trial design can be further improved to account for this issue by using a platform or “flip-flop” design [5] of multiple TiTE-CRMs. Alternative design methods for dealing with late-onset toxicities have also been proposed, which are beyond the scope of this paper [34–38].

## Conclusion

As model-based phase I designs are being more widely used and discussed, we hope that this paper will help clinical trial teams to understand TiTE-CRM and its nuances, and provide a starting point for using this methodology in practice. Some of the information and advice given in this paper will also likely be relevant for other time-to-event phase I designs.

We have found that designing and running early-phase trials using the TiTE-CRM can be complex, but is feasible and worthwhile. By sharing experience and knowledge, we aim to demystify the conduct of dose-finding trials using TiTE-CRM methodology.

## Supplementary information

**Additional file 1.** Dose Calculation for a TITE-CRM Study

## Data Availability

Not applicable.

## Abbreviations

CRF: Case Report Form
CRM: Continual Reassessment Method
DLT: Dose Limiting Toxicity
MTD: Maximum Tolerated Dose
SDV: Source Data Verification
SOP: Standard Operating Procedure
TiTE-CRM: Time-to-Event Continual Reassessment Method
TTL: Target Toxicity Level
