# Supplementary material for: Practicalities in running early-phase trials using the time-to-event continual reassessment method (TiTE-CRM) for interventions with long toxicity periods using two radiotherapy oncology trials as examples
Source: BMC Med Res Methodol. 2020 Jun 22;20:162. doi: 10.1186/s12874-020-01012-z (PMC7477911; doi:10.1186/s12874-020-01012-z)
Supplement: Supplementary file 1 — Additional file 1. Dose Calculation for a TITE-CRM Study [file 12874_2020_1012_MOESM1_ESM.pdf]

Dose Calculation for a TITE-CRM Study

Preparing the input data set

This template uses an Excel dataset, saved as a plain-text comma-separated value (.csv) file. The file should have the following columns:

- patient id
- dose level
- start treatment date
- end treatment date
- dropout date
- followup date
- DLT date

The R code below reads the dataset from a Excel csv file and prepares it to use as model input:

```
file.date <- "20190101" # the idea is to have separate directories for each calculation
                        # so that old data is never lost
file.name <- file.path(".", "data", file.date,
                       paste("patientdata_", file.date, ".csv", sep=''))
patient.data <- read.csv(file=file.name, header=TRUE)
```

The date of the Excel file with follow-up data was 01 January 2019.

```
## check the date formats of the input file and change if necessary
patient.data$start.treatment.date <- as.Date(
  patient.data$start.treatment.date, format="%d/%m/%Y")
patient.data$end.treatment.date <- as.Date(
  patient.data$end.treatment.date, format="%d/%m/%Y")
patient.data$followup.date <- as.Date(patient.data$followup.date, format="%d/%m/%Y")
patient.data$DLT.date <- as.Date(patient.data$DLT.date, format="%d/%m/%Y")
patient.data$tox <- as.numeric( !is.na(patient.data$DLT.date) )
```

```
## calculate follow-up duration in days
patient.data$start <- patient.data$start.treatment.date
patient.data$stop <- pmin(patient.data$followup.date,
                          patient.data$DLT.date, patient.data$dropout.date, na.rm=TRUE)
patient.data$followup <- as.numeric(patient.data$stop -
                                      patient.data$start) # follow-up in days
if(any(is.na(patient.data$followup))){
  stop("The follow-up time could not be calculated for one or more pateints")
}
```

The code below shows the data that was used:

```
options('width'=120) # set line width to make the output fit on the page
print(patient.data[, c('dose.level', 'tox',
                       'start.treatment.date', 'followup.date', 'DLT.date',
                       'stop', 'followup')])
```

```
##   dose.level tox start.treatment.date followup.date   DLT.date       stop followup
## 1          1   0           2018-01-01    2019-01-01       <NA> 2019-01-01      365
## 2          2   0           2018-05-01    2019-01-01       <NA> 2019-01-01      245
## 3          3   1           2018-08-01    2019-01-01 2018-09-01 2018-09-01       31
## 4          2   0           2018-09-01    2019-01-01       <NA> 2019-01-01      122
```

Setting parameters and choosing a weight function

```
prior.dlt <- c(0.05, 0.10, 0.20, 0.30) # prior parobabilities for dose level 1,2,3,4
dosename <- paste("dose", as.character(seq(1, length(prior.dlt))), sep=" ")
target <- 0.20 # The target probability of toxicity at the MTD

t.acute <- 3*30 # acute DLT period (counting time from start of treatment)
t.late  <- 365  # time unitil end observation late-onset toxicity
```

The following weight function was chosen to account for the proportion of the observation period that each currently enrolled patient has been observed. It is linear in the follow-up time t until the end of the observation window:

$$w(t) = \begin{cases} 0 & \text{if } t < 0 \\ \frac{t}{T} & \text{if } 0 \leq t < T \\ 1 & \text{if } T \leq t \end{cases} \tag{1}$$

with $T = 365$ days. An implementation of the function in R code is given below:

```
getWeight <- function(t, t.late){
    y <- rep(NA, times=length(t))
    y[t < 0 ] <- 0
    y[0 <= t & t <= t.late] <- t[0 <= t & t <= t.late ]/t.late
    y[t.late < t] <- 1
    return(y)
}
patient.data$weight <- getWeight(patient.data$followup, t.late)
```

An example of a weight function that takes into account an acute DLT period and a late-onset DLT period is given below. It gives half the weight to the acute DLT period and half to the remainder of the observation time window:

$$w(t) = \begin{cases} 0 & \text{if } t < 0 \\ \frac{t}{2t_{\text{acute}}} & \text{if } 0 \leq t < t_{\text{acute}} \\ \frac{1}{2} + \frac{(t - t_{\text{acute}})}{2(t_{\text{late}} - t_{\text{acute}})} & \text{if } t_{\text{acute}} \leq t < t_{\text{late}} \\ 1 & \text{if } t_{\text{late}} \leq t \end{cases} \tag{2}$$

with $t_{\text{acute}} = 90$ days and $t_{\text{late}} = 365$ days.

```
# getWeight <- function(t, t.acute, t.late){
#     y <- rep(NA, times=length(t))
#     y[t < 0 ] <- 0
#     y[0 <= t & t <= t.acute] <- t[0 <= t & t <= t.acute ]/t.acute*0.5
#     y[t.acute < t & t <= t.late] <-
#       0.5 + (t[t.acute < t & t <= t.late] - t.acute) / (t.late - t.acute)*0.5
#     y[t.late < t] <- 1
#     return(y)
# }
# patient.data$weight <- getWeight(patient.data$followup, t.acute, t.late)
```

If you want to use this function, remove the # symbols. (The # symbol inside a chuck of code marks the rest of the line as comment.)

Running the model

The following code runs the model and shows its standard output:

```
library(dfcrm)
# If you get error:  there is no package called 'dfcrm'
# then you need to run the command: install.packages('dfcrm')
# (Needs to be run only once. )

m.dlt <- titecrm(prior = prior.dlt,
                 target = target,
                 tox = patient.data$tox,
                 level = patient.data$dose.level,
                 weights = patient.data$weight,
                 pid = patient.data$patient.id
                 )
print(m.dlt)
```

```
## Today:  Mon Aug 26 15:04:49 2019
## DATA SUMMARY (TITE-CRM)
## PID   Level   Toxicity   f/u    Weight      Included
## 1     1   0       N/A     1       1
## 2     2   0       N/A     0.671      1
## 3     3   1       N/A     1       1
## 4     2   0       N/A     0.334      1
##
## Toxicity probability update (with 90 percent probability interval):
## Level     Prior   n   total.wts   total.tox   Ptox    LoLmt   UpLmt
## 1     0.05    1   1       0       0.178   0.006   0.562
## 2     0.1     2   1.005       0       0.265   0.019   0.642
## 3     0.2     1   1       1       0.396   0.062   0.734
## 4     0.3     0   0       0       0.5     0.125   0.793
## Next recommended dose level: 1
## Recommendation is based on a target toxicity probability of 0.2
##
## Estimation details:
## Empiric dose-toxicity model: p = dose^{exp(beta)}
## dose = 0.05 0.1 0.2 0.3
## Normal prior on beta with mean 0 and variance 1.34
## Posterior mean of beta: -0.552
## Posterior variance of beta: 0.444
```

The standard output above may look a bit cluttered. A summary table of the results is shown below:

Table 1: Model results

Dose level	Prior	Updated estimate	Lower limit of 90 % CI	Upper limit of 90 % CI
1	0.05	0.18	0.01	0.56
2	0.10	0.27	0.02	0.64
3	0.20	0.40	0.06	0.73
4	0.30	0.50	0.13	0.79

The next recommended dose based on the model alone was 1. This is the dose level with probability of DLT closest to the target of 0.2 (so it could be greater than the target).

Checking escalation restrictions

```
## Example: do not allow skipping of any dose level
max.dose.level <- max(patient.data$dose.level)
next.allowed.dose <- max.dose.level + 1

last.patient.dose <- patient.data[order(patient.data$start.treatment.date),
                                  'dose.level'][nrow(patient.data)]

## Test restrictions of sufficient follow-up in previous dose levels
## Example: at least 3 months (90 days) of follow-up must be observed
##          from at least three patients in the previous dose level
minimal.followup <- 3*30 # three months = 90 days
min.followup.n.patients <- 3

n.patients.with.followup <- nrow(subset(patient.data,
                                        dose.level == max.dose.level &
                                        followup > minimal.followup) )

if(min.followup.n.patients <=  n.patients.with.followup){
  escalation.allowed <- TRUE
} else{
  escalation.allowed <- FALSE
}


if(escalation.allowed){
    next.dose <- min(m.dlt[['mtd']], next.allowed.dose) # it's OK to go up
} else{
   next.dose <- min(m.dlt[['mtd']], max.dose.level) # do not go up
}
```

To determine the dose for the next patient, the following was taken into account:

- the highest dose level at which a patient was treated so far was 3;
- the MTD estimated by the model was 1;
- that means that no escalation is recommended.

Therefore, the next recommended dose level is 1.
